# Supplementary material for: Pilot Study: Safety and Performance Validation of an Ingestible Medical Device for Collecting Small Intestinal Liquid in Healthy Volunteers
Source: Methods Protoc. 2024 Feb 4;7(1):15. doi: 10.3390/mps7010015 (PMC10892249; doi:10.3390/mps7010015)
Supplement: Supplementary file 1 [file mps-07-00015-s001.zip › mps-2801689-supplementary.pdf]

Supplementary Tables

Table S1: Exclusion criteria for the clinical investigation.
Lactose and fructose intolerance,
Subjects with a history of gastroparesis,
Subject having had a colonoscopy preparation up to 1 month prior to inclusion,
Subject in a period of exclusion from another clinical investigation,
Subject receiving more than 4500 euros in compensation for participation in other biomedical research in the 12 months preceding this clinical investigation,
Subject who cannot be contacted in an emergency,
Subject who, during the 15 days prior to inclusion, has taken and/or is scheduled to take any of the following treatments during the study: antidepressants, anxiolytics, antiparkinsonian agents, neuroleptics, antibiotics and prebiotics,
Subjects who, during the previous 7 days, have taken at least one treatment for irritable bowel syndrome (antispasmodics, etc.), constipation (laxatives) or diarrhea,
Subjects scheduled for colon surgery,
Subjects with hepatic, cardiac, congenital or renal comorbidity,
Subject with long-standing diabetes,
Subjects undergoing radiotherapy or taking long-term anti-inflammatory drugs,
Subject with gastric fullness,
Subject with known or suspected obstruction of the gastrointestinal tract, including known intestinal or intestinal inflammatory diverticula (folds),
Presence of swallowing disorders or Zencker diverticulum,
Nausea, deviated nasal septum,
Subjects with contraindications to esophageal tube placement: people with chronic esophageal pathology (e.g., treated gastroesophageal reflux disease, hiatal hernia),
Drug or alcohol abuse (3 units for men and 2 units for women according to WHO criteria),
Known allergy to silicone or iodine,
Subject refusing medical or surgical imaging in the event of non-recovery of the module,
Persons covered by articles L1121-5 to L1121-8 of the French Public Health Code (pregnant women, parturient, nursing mothers, persons deprived of their liberty by judicial or administrative decision, persons under psychiatric care and adults under legal protection or unable to express their consent).
Absence of highly effective contraception for women capable of childbearing until the end of the cycle following the end of participation in the study.
